# The Hospital. Nursing Section

**Published:** 1904-08-13

**Authors:** 


### The Hospital

**Nursing Section.**

**Contributions for this Section of "The Hospital" should be addressed to the Editor, "The Hospital" Nursing Section, 28 & 29 Southampton Street, Strand, London, W.C.**

No. 933.—Vol. XXXVI. *SATURDAY, AUGUST* 13, 1904.

### Notes on News from the Nursing World.

### THE ROYAL VISIT TO HASLAR HOSPITAL.

The visit of the King and Queen and the Prince of Wales to the Royal Naval Hospital at Haslar on Monday was a pleasant surprise to the nursing staff. They had for some time been looking forward to the opportunity of welcoming her Majesty, who, as President of Queen Alexandra's Royal Naval Nursing Service, naturally takes the greatest interest in the members who are on duty at the principal naval hospital. Miss Cadenhead, head-sister, accompanied the medical officers with the Royal party through the wards. Both she and the whole of the nursing staff had the honour and gratification of being introduced to their Majesties. One of the sisters writes that she was just rushing to her cabin to fetch her sterilised dressing tins for an operation at 11 A.M., when, passing her ward, she saw coming from the far end a whole bevy of officers in full naval uniform, headed by the King. For a moment she gazed in amazement, then she darted to her cabin and donned her cape and cuffs, and walked down the ward to meet their Majesties. The King, in animated conversation with two medical officers, saluted her, and she hastened to meet the Queen, who graciously shook hands with her. Her Majesty said what a delightful ward it was, and how glad she was to be able to pay a visit to the hospital; she then moved to the bedside of a patient, inquiring what was the matter with him, and if he was getting better, etc. Indeed, for each patient she had a word and a smile; one man, with a bad knee, she was particularly interested in, but the sister assured her that he was much better and discharged to duty for the following day. Their Majesties visited several other wards and signified satisfaction with all that they had seen.

### THE QUEEN AT NETLEY HOSPITAL.

On Thursday last week the Queen, accompanied by the Prince of Wales and Princess Victoria, visited the Royal Victoria Hospital, Netley, for the purpose of inspecting the new quarters of Queen Alexandra's Nursing Service. The Royal party were conducted over the quarters and through other parts of the building by Miss Addams-Williams, the lady superintendent. The visit lasted over an hour, and the Queen manifested the greatest interest in all the arrangements for the comfort and convenience of her nurses.

### THE AUSTRALIAN ARMY NURSING SERVICE.

It is officially announced that the Australian Army Nursing Service will consist of six lady superintendents, six matrons, and 96 nursing sisters. The New South Wales Contingent was formed before the outbreak of the war in South Africa, and the Victoria Contingent, with a lady superintendent, a matron, and 24 sisters, has just been inaugurated. The number of members and the regulations are the same as those of the New South Wales State; but the States of Queensland and South Australia will only have 14 sisters, and the States of Western Australia and Tasmania 10. Colonel Ryan, in a lecture delivered to the nurses of the Victoria Service at the time of inauguration, devoted a good deal of attention to the question of uniform, which will be identical in every State, and bears a striking family resemblance to that worn by members of Queen Alexandra's Imperial Military Nursing Service. In fact, the only difference is that the facings are chocolate colour instead of scarlet.

### THE UNJUST MATRON.

The matron of the Royal South Hants and Southampton Hospital protests against the proposal that the power of discharging a probationer should be, in all cases, taken out of the hands of the matron, and vested in the hospital committee. Miss Mollett thinks that as the matron engages the nursing staff, subject to rules approved by the committee, it would be curious if she were denied the right of reducing her staff of those members who are either incapable of doing their duty properly, or who are dangerous to the good tone and discipline of the nursing-school. Of course, the difficulty is the unjust matron, but as we believe her to be the very rare exception, and so long as the right of a discharged probationer to appeal to the committee is carefully guarded, we think that the power of discharge should rest with the head of the nursing school. If a woman cannot be trusted to treat the nurses under her fairly, she is not fit for the position of matron. In these days, when the fierce light of publicity beats upon everything, the unjust matron can easily be brought to book, and her possible existence is not a sufficient reason for depriving the just matrons of an obligation which undoubtedly helps them to maintain discipline and to exercise authority.

### THE BUTE TRUSTEES GIVE WAY.

It is extremely satisfactory to learn that the trustees under the will of the late Marquis of Bute have recognised the reasonable attitude of the committee of the Seamen's Hospital at Cardiff, and have withdrawn the stipulation that, in order to profit by Lord Bute's bequest, the nursing must be entrusted to a Roman Catholic sisterhood. They now only require that care shall be taken that Roman Catholic patients shall be attended by Roman Catholic nurses, and as the committee were always willing to make this arrangement, the controversy which at one time threatened to seriously cripple a most useful institution, may be regarded as closed. We do the trustees the justice of supposing that they only pressed the point because the terms of the will are that "if possible" the nursing should be done by Roman Catholics; and that they have given up the contest because they found that the difficulty of carrying out the wish expressed was insuperable.

### MIDWIFERY NURSING IN POOR-LAW INFIRMARIES.

The Kensington Guardians have assented to a recommendation of the Infirmary Committee that application be made to the Local Government Board for the appointment of two midwifery nurses at a salary of £34, rising to a maximum of £38 per annum. Miss Alexander characterised the proposal as a very retrograde movement, but she did not bring forward an amendment. We do not think that she is right. The proposal is due to the advice of Dr. Potter, the medical superintendent of the infirmary, who, as an appointment was about to be made in the maternity wards, recommended that in future instead of the staff comprising midwifery nurse and an assistant midwifery nurse, it should consist of two midwifery nurses, doing duty alternately by day and night for periods of three months. We entirely agree with Dr. Potter that the nightly rest of the midwifery nurse ought not to be broken, and that no nurse ought to be expected to do duty both by day and night. Moreover, a system similar to that now suggested has been carried out in several workhouse infirmaries to which maternity departments are attached, and, as Dr. Potter says, appears to be working satisfactorily.

### PROBATIONERS AT NORFOLK AND NORWICH HOSPITAL.

The Board of Management of the Norfolk and Norwich Hospital have, we understand, decided to discontinue the present system of requiring probationers to pay a premium. In future, each probationer will be required, at the end of two months' trial, to enter into an agreement for three years and further to serve for a fourth year on the private nursing staff, provided the nursing committee shall then consider her eligible. A salary of £8 will be paid for the second year, £16 for the third year, and £24 for the fourth year. Accommodation for the nursing staff is provided by the Leicester Nurses' Home, adjoining the hospital, which was opened in July last year.

### THE EXTRA DUTIES OF A POOR-LAW MEDICAL OFFICER.

The Scarborough Guardians, instead of asking the Local Government Board for instructions in respect to the claim of Dr. Foley for fees amounting to £10 for the examination of candidates for the position of nurse, have elected to be guided by the advice of their own house committee, who recommended that the reduced sum of £3 3s. should be paid. The clerk to the board advised that Dr. Foley had no claim at all for remuneration, but he also stated that the board had no claim on Dr. Foley's services for attending to the officers of the board. One of the guardians said that before the medical officer was called to examine the candidates, the question of remuneration should have been raised, but this contention would have had more force if another guardian had not rejoined that the practice had been going on for six or seven years. We still think that the issue should have been referred to the Local Government Board. It is possible that, in existing circumstances, the amount awarded by the guardians to Dr. Foley may be surcharged.

### NURSES AND POLITICAL INFLUENCE.

An attempt is being made in Australia to foster among nurses the idea that they ought to adopt the principles of trade unionism. We are glad to see, however, that it has not met with much encouragement. The general feeling among trained nurses appears to be that they do not require any political influence exerted on their behalf, simply because, as one of their number admirably puts it, "the long hours and the hard work inseparable from private nursing is their willing and cheerful contribution to the cause of suffering humanity, and is not in any sense exacted from them." The same nurse says, "the Trade Hall spirit of measuring out so many hours and so much exertion in each day, regardless of the public need, has so far found no favour among our nurses, and it will be an evil day for profession and public when it does so." This is the true nursing spirit, and may be commended to discontented hospital probationers, whether in England or the colonies, who think that it is possible to be loyal to a noble avocation and yet make purely selfish purposes the primary object in view.

### SUPERANNUATION OF A SISTER.

A very proper course has been pursued by the Croydon guardians in the case of Miss Pope, the late head nurse and ward sister, who, at the age of 45, has become disabled through acute rheumatism from following her employment. Miss Pope has actually completed 14 years of service under the Local Government Board, and the Croydon guardians have decided for superannuation purposes to add six years to the period. It is a matter for regret that an excellent nurse should have been compelled to terminate her career prematurely, but it is satisfactory to find that her work has been appreciated.

### TRAINING FOR MIDWIVES AT CARDIFF.

The importance of local action in connection with the Midwives Act is fully recognised in Cardiff, and there will shortly be a training centre for midwives in full working order in the town. The committee of the local branch of the Victoria Jubilee Institute will open a maternity department in October, and as it is calculated that no fewer than 3,000 confinements are annually attended by midwives in the county borough of Cardiff, the field of work is likely to eventually become a large one. A special committee has been appointed, and they have drafted a set of rules which they believe will both safeguard the charity from abuse, and will also secure the support and co-operation of the local medical profession. It is estimated that the scheme will involve an annual expenditure of about £400, as well as a considerable initial outlay; but we are glad to hear that the committee are confident that an appeal to the generosity of the well-to-do will not be made in vain.

### A COUNTY ASSOCIATION FOR HEREFORDSHIRE.

Under influential auspices a conference has been held at the Shire Hall, Hereford, for the purpose of considering the formation of a county nursing association affiliated to Queen Victoria's Jubilee Institute on the lines described in detail in our issue of last week by Miss Amy Hughes. Among the speakers were Mrs. Barneby of Longworth, Lady Biddulph of Ledbury, Mrs. Percival and Miss Hughes. A resolution in favour of the object was carried unanimously, and subsequently it was decided to endeavour to arrange with the County Hospital for the supply, when necessary, of emergency nurses to affiliated associations. It was announced that at present the donations for the reserve fund amounted to about £114, while about £60 had been promised in yearly subscriptions. The conference consisted entirely of ladies, but one of the speakers paid a vigorous tribute to Mr. Moody of Breinton, of whom she said that he had really given himself up to the work, and she wished him to understand that the reason he was not invited that day was simply because he belonged to the male sex. The Executive Committee is composed of ladies, but a man has been appointed honorary treasurer.

### MAJOR BALLANTINE'S RETIREMENT.

The excellent work done by Major Ballantine, who has just ceased to be master of Manchester Workhouse, deserves to be warmly acknowledged. The ability, the knowledge, and the judgment of the gallant officer who, for 27 years, was the principal official at Crumpsall, has contributed, in no small degree to the success of a training-school which is second to none among Poor-law Infirmaries. He was always ready to co-operate with the lady superintendent in the promotion of reforms, and never lost an opportunity of showing that he had the interests of the nurses at heart. He will be much missed by every member of the staff.

### THE NEW SCHEME AT CAMBORNE.

At the annual meeting of the Camborne District Nursing Association reference was made to the new scheme by which the nurses are permitted to attend persons in better circumstances on payment of a weekly sum. This is placed to the credit of the Association, and the report shows that in the year just completed the amount received from these patients was £8 16s. From all sources the receipts, including a balance brought forward of £141, a subscription of £4 4s. from the Redruth Guardians, street collection £18, receipts at football matches £16 19s., amounted to £288 15s. 1d., and the expenditure of the year just left the balance intact. The question of employing a third nurse was again raised, and a proposal to ask the Urban District Council to consider the matter was agreed to. The point was referred to the Urban District Council on the ground that in a year or two an Act of Parliament will come into force which makes it compulsory for some local authority to provide for nursing, etc.

### NURSES' SUCCESS AT ASHTON-UNDER-LYNE INFIRMARY.

Charge Nurse Kershaw and Probationer Nurses Crossland, Etchells, and Mather, of the Ashton-under-Lyne Workhouse, were successful in obtaining the London Obstetrical Society's certificate at the recent first examination of nurses from this institution. The result is very creditable to Miss Tattersall, who has only been superintendent of nurses at the Union Infirmary for the past eight months. She has specially coached the nurses, and taken a keen interest in their welfare.

### PROGRESS AT BURSLEM.

In the report for 1902 of the Burslem District Nurses' Institution and the Alcock Memorial Convalescent Fund, the committee had to deplore a considerable falling-off in subscriptions. The report for 1903, which has just been issued, shows a very marked improvement. Several arrears were paid during the year, and a number of new subscriptions and donations were received, including a donation of £6 13s. 4d. from the Cobridge Bowling Club, being one-third of the proceeds taken at their flower stall on Hospital Saturday. In fact, altogether the receipts amounted to £171 14s. 10d., as against expenses £147 17s. 0d., a very satisfactory result. The number of visits paid by the two nurses was 4,422, and the number of cases attended 278.

### DISTRICT NURSING AT WESTHOUGHTON.

In their annual report the Committee of the Westhoughton Cottage Hospital and Sick Nursing Association acknowledge in a handsome manner the services rendered by the district nurse, who attended 301 cases and paid 6,091 visits. We are not surprised that, in these circumstances, the importance of appointing a second nurse is recognised, especially as the area the single nurse has to cover is large. As the financial position of the organisation is satisfactory, we hope to learn before long that she has been assigned a colleague.

### AN INVENTION BY AN AUSTRALIAN MATRON.

Miss Evelyn Conyers, whose appointment as matron of the Fever Hospital, Melbourne, was announced by us last week, lately patented in Australia an improved supporting frame to be employed with a slipper bed-pan. We understand that the invention has already been used with considerable success in various Colonial hospitals.

### SHORT ITEMS.

The King has sanctioned the appointment of Princess Alice Marie Victoria Auguste Pauline, of Teck, and Viscountess Galway as ladies of justice of the Order of the Hospital of St. John of Jerusalem in England, and of the Countess of Dudley as a lady of grace.—The matron of St. Mary (Islington) Infirmary, having stated that Miss Little, whose appointment as sister at Fulham Infirmary a few weeks ago was announced, had not been trained at that institution, we are informed that Miss Little was trained at the old infirmary at Islington and not at the new one on Highgate Hill.

### The Nursing Outlook.

"From magnanimity, all fear above;

From nobler recompense, above applause,

Which owes to man's short outlook all its charm."

### THE GUILD OF ST. BARNABAS.

The religious spirit in nursing life, both in England and America, is best seen in the quiet progress of the Guild of St. Barnabas.

The Guild was founded in 1876 by Miss Antrobus, a former night-superintendent at the Middlesex Hospital, with the Rev. E. F. Russell, of St. Albans, as chaplain. The object of the Guild is both religious and social: it has its offices in Brook Street, Holborn, and it issues a little monthly paper called *Misericordia.* Its members are bound together by daily prayer and a common "rule," which is an aid in the spiritual trials peculiar to a nurse's life. But there is also a monthly service held at St. John's Church, Red Lion Square, and after this special service the members generally adjourn to the crypt for tea and talk, and the social side of the Guild is brought to the fore. There are local branches in many of the large towns, and the total membership of the English Guild is over 2,000. In America the Guild was founded by the Rev. Father Osborne, of Boston, about 1887: it has branches in most of the big cities; and the secretary-general is Mrs. William Reed Howe, 252 Main Street, Orange, N.J. Its object is also religious and social, and it works by means of the daily rule of life, the monthly service, the monthly meeting, and the annual festival. According to an official account—

It is intended to help the nurse to remember her high calling as a follower of Him who "went about doing good," a sharer in His work of ministry and love. It is intended to encourage her to continue bravely in the work of comforting, healing, saving, giving rest, and manifesting in herself that sweetness, patience, and unwearying labour which were characteristics of His earthly life. It is her privilege also to be a sharer in His life of toil and sorrow.

The name of St. Barnabas is chosen for the Guild because he followed in the footsteps of his Master, and those who minister to the sick and suffering are rightly reckoned as the companions of the "Son of Consolation." The Guild prayer daily asks for the same spirit of consolation, "that by gentleness and love, in faithfulness and patience, we may serve God's afflicted children."

The American membership is also about 2,000. A very practical side of the organisation on both sides of the water is a strong interest in mission work and an endeavour to supply suitable nurses for the mission field. The English Guild has supplied most of the nurses for the Universities' Mission to Central Africa; the American Guild has supplied nurses to Alaska, China, and the Philippines. When American members are on this side they are always warmly welcomed to the services and meetings at St. John's; and at last year's festival, at which the Bishop of London preached, Bishop Whitehead, the chaplain-general of the American Guild, was present. The numbers of the American Guild have greatly increased since they dropped their little *News-Letter* and made the *American Journal of Nursing* their organ for spreading information. We believe that a like move would be beneficial here were it not for the distressing tone of antagonism kept up by the strife for power in nursing politics. Nothing could be more contrary to the charity and humility of the daughters of consolation than the unkindness and carping which mark so much of modern nursing journalism.

And such a Guild, binding together the members of the same Church and the same profession in a still closer bond of sympathy and fellowship, is very helpful to a nurse. There is no one who lives a more lonely life than the average private nurse: she is at the beck and call of others; she has no home of her own; she is continually making new acquaintances, trying to adapt herself to new circles and new conditions. In the midst of this ever-changing and most distressing outward life to have an inward life of peace and fellowship to which she can retire for rest and refreshment of soul is comforting and sustaining. There is a practical side also. How quickly does the feeling towards one's fellow-nurse become friendly if one sees the little green manual on her table, or notices the bronze medal on her watch-chain! How helpful to know that there is always the superior and the chaplain to turn to in either bodily or spiritual trouble—that there is an ear open to our story, a heart open to our grief, and that even temporal assistance can be secured from the Guild! In America there is a special committee for organising the benevolent side of the Guild and helping those who cannot afford to finish their professional training, or who are sick and laid up, or otherwise in distress. And in travel one secures friends everywhere by means of the fellowship of the Guild. To every line of life there are special difficulties and temptations, and it was the knowledge of this fact which led in mediæval days to the establishment of these workers' "guilds." But nothing could be more disastrous than for any nurse to join a guild for the sake of practical advantages. Spiritual integrity is one of the great needs of the present day, and to be true to the faith or the doubt that is in us seems equally impossible to many lukewarm natures. For those in whom the ecclesiastical spirit is not strong a guild with such a rule of life as that of St. Barnabas is not intended. But to those nurses who are in sympathy with High Church methods the Guild offers help and encouragement which they would be wise to accept.

### Lectures Upon the Nursing of Mental Diseases.

By ROBERT JONES, M.D.Lond., B.S., F.R.C.S.Eng., M.R.C.P.Lond., Resident Physician and Superintendent of the London County Asylum, Claybury.

LECTURE XVI. (*continued from p.* 247).

In every institution for the insane it is the rule to allow as much of the patients' wishes in all things to be granted as may be safe and possible. No one knows who has not experienced service among the insane the trouble, anxiety, and worry caused by the constant buttonholing and references made by cases of religious insanity to the safety of others and others' souls. When service is over these and other patients are counted, collected, and taken out for exercise into the various gardens or, as they are often called, exercising or airing courts. Those patients who for any reason—noise, excitement, or restlessness—are yet in their rooms are now dressed, and in fine weather are also taken out into the airing courts, as the wards are now free and supervision is undisturbed. During the time the patients are in the airing courts the nurse sees that they do not lie down on the gravel or paths or walks, nor on the grass in damp weather. In very hot weather they must be placed in the shade, and in very cold weather be encouraged to take brisk walks, or to take part in some outdoor amusement, such as skipping, looking after birds or animals, such as guinea pigs, tame mice, or rabbits, which are a special feature of some institutions. By 10 o'clock the cleaning of the dayrooms, bedrooms, and passages is to be completed; no mops, pails, brushes, steps, or rubbish must be left about; the utmost cleanliness is to be observed, and the nurses are to be neatly dressed and everything ready for the visit of the medical officer, a full report being made to him when on his round—as fully described in a previous lecture. The sick diet lists are placed out ready for daily inspection by the medical officer, who also revises the medicines, the suicidal "parchments," and inspects the wards, bath-rooms and lavatories. About half an hour before the patients come in from the airing courts, the more troublesome, excited or noisy ones are collected, as also those with suicidal tendencies, and they are either securely brought in and placed in the wards, or they are again undressed and placed in their rooms until they go out again after dinner, which meal they may take in their own rooms. During the morning the tins and trays are taken down to the kitchen for dinner and are fetched therefrom on trollies shortly after noon with the dinners for the wards. In many institutions the patients dine in large halls, either one or both sexes together. The nurse must be careful in supervising the diets of patients—each of whom receives what is allowed according to a scale fixed by the committee of the asylum if a public one. Every patient may, if he or she desires it, see the diet weighed out for him or herself, but it is usual to place the portion of meat allowed on the weighing scale or balance, and the nurse with this before her serves out the rest upon this pattern. In the case of "suicidal patients" the meat and vegetables are cut up beforehand and the patient uses a spoon and fork at a separate table. The knives in an asylum are specially constructed so as to allow of a sharp edge on about an inch of the knife, and also about an inch from the point, in order to avoid any possibility of injury or harm to himself or others, should any tendency in this direction arise. The patients, even the excited ones, are more tranquil after than before dinner, owing to a physiological withdrawal of the blood from other organs to the digestive tract. In this way half the nurses in a ward are permitted to go to their own dinners when the patients have been served, the other half similarly dining after their return. In some institutions the nurses may have their breakfast and tea in the wards, dining only in a separate mess-room at a general mess-table. In others they have all their meals in a separate mess-room, breakfasting before they go on duty in the morning. These are matters of administration which vary in different institutions, and each method has its champions. At half-past two the patients are again collected for airing court, and they go out until four o'clock, the excited and agitated ones going out as in the morning after the others, and coming in before them. In the afternoon the quiet, orderly, and convalescent patients or those who are chronic but tractable and express the wish to go, are taken, either into the grounds, or beyond and outside the grounds and into the adjoining village or neighbouring town—if not too distant—in order to "shop" or make little purchases for themselves, the nurses personally supervising their marketings with a not too direct attention, but with a sufficiently close guard to see that the confidence reposed in them is not betrayed.

Care must always be taken that patients who walk along the streets or roads are safe from the traffic, that they are not in the way of the neighbours, for the law is strict as to the conduct of insane patients when exercising beyond their own grounds, and any complaint from others not connected with the asylum is sufficient to prevent the enjoyment of this liberty, which is so greatly prized in asylums and which is so beneficial a remedy in the treatment of the insane incarcerated in institutions and separated from their friends and ordinary life outside. From 5 to 5.30 o'clock comes tea, being preceded by that for the nurses. After each meal the cups, saucers, plates, dishes, etc., are to be carefully washed and put away, and every knife, fork, and spoon used at meals by patients is carefully counted over and examined before patients rise from table, so that the numbers are correct. They are then carefully locked up in a special box for the purpose. The loss of a knife or fork after a meal is a most disturbing factor in an asylum and means a great upset, as every patient is personally searched, the whole of the ward overturned, every sink and trap examined, and no refuse leaves the ward nor does any patient go out to exercise, or chapel, or entertainments until the missing knife or fork is found. After tea in some institutions evening chapel takes place and patients are always allowed the use of hymn books and prayer-books when so wishing. In the summer during fine weather what is called "evening airing court" is also arranged, or the patients walk out into the grounds, or into the cricket-field and associate in games, dances, listening to music, or watching cricket or croquet matches. They are always encouraged either to take part in some diversion or to be occupied at needlework or in some other congenial pastime.

At seven o'clock all patients are in their wards, except on entertainment nights, and about this hour the extras ordered from the kitchen for the different wards are fetched. These are for the use of patients during the night, and are served by the night nurse. They are generally beef-tea, mutton-broth, sago, arrowroot, gruel, and lemonade.

From half-past seven to a quarter to eight the patients prepare for bed. They have medicines or special draughts ordered by the medical officer served out to them about this time. Their day-clothes are wrapped up and placed in bundles on racks, each patient's wardrobe compartment being labelled with her name or the number of her bed, and her clothes placed thereon. In the case of patients suspected

### LECTURES UPON THE NURSING OF MENTAL DISEASES—*Continued.*

of dangerous propensities, the day clothing is carefully searched for any weapon of offence, and she herself is examined every night in case she may have secreted something about her person which may prove to be dangerous to herself or others. These precautions are fully detailed in a previous lecture. The tobacco pipes of the male patients are given up voluntarily before bed time and placed by the patient or his attendant in a special rack, with a number or some other means of identifying them afterwards, and the bundles of clothing are placed in a like manner upon racks on a concrete or stone floor as a precaution against the spread of fire, such as has occurred when a lighted pipe was placed in a patient's pocket and overlooked.

A list of all the patients sleeping in a ward is made out, with the names, number of room or bed occupied, and notes are entered for the night nurse, in regard to any one requiring special attention. The charge nurse or sister personally hands her cases over to the night nurse, and states that she has done so in her report for the day, which she makes up before leaving duty, and hands this report, called the Ward or Day Report, to the matron or her deputy.

The charge nurse once a week prepares a list of all stores and articles—furniture, utensils, etc.—and submits the list to the matron, who further requests the steward to supply the same. Her list includes the number of boots and shoes to be repaired, and on certain specified days the laundry is to be seen to and clothes which need it are to be listed for condemning, which is done by the matron herself or by the "work mistress," or some other official delegated for the purpose; "condemned clothing" has to be replaced, so that the inventory of all articles kept in the ward is correct, and for which the charge nurse is held responsible.

The night nurse comes on duty at a quarter to eight, and remains so until half-past six the next morning. She first takes over all the patients from the day nurse, seeing each individually, and then examines her list of patients, more especially noting those who require attention, food, cordials, or medicines during the night, and ascertains if everything necessary has been left out for them. The night nurses in asylums are either attached to a ward or they promenade through certain sections, these latter being denominated "patrol" night nurses. These visit some of the wards several times during the night, administer medicines and nourishment as directed, and pay proper attention to such patients as are restless or of defective habits (*i e.* are wet or dirty), who wish for water to drink or who need change of dress. Special attention is paid to the cleanliness of wet or dirty patients, who usually have "mackintosh sheets" below the bottom blanket to save the bed or mattress from being soiled. In going through the wards at night care must be taken to avoid disturbing the patients. Soft shoes are provided and must be worn by the night nurse, the doors must be locked and unlocked as quietly as possible and a light is to be carried in lanterns provided, so as to avoid turning the electric or gas-light fully on in the dormitory. The proper clothing of patients who refuse to lie in bed must be carefully attended to, and their feet must be protected. Patients who are disposed to suicide, or who are subject to epileptic attacks, or who have a habit of lying on their faces, sleep in special dormitories where there is a constant night nurse on duty. These must be especially watched and are called "observation" cases. Such are those already named and all newly admitted cases, as also those who are seriously ill, who suffer from bodily as well as mental ailments and who constitute the ordinary medical cases of our hospitals. In case of any accident or unusual circumstance the night nurses shall at once summon the aid of the medical officer. In the morning they call up the day attendants at the usual hour of rising, and they hand to these their patients as already described. When this has been done, and their reports are completed and handed to the matron or her deputy, their duties cease. The following is a copy of the night nurse's report used in Claybury Asylum:—

### Night Nurse's Report.

Ward ..................

FEMALES. Date .................. 190 .

I have taken over ........... patients sleeping in ........... dormitory of Ward ...............

(Signed) ........................

| Ward. | Patients' Names. | Remarks. |
|---|---|---|
| | | |

| Clothes sent to laundry. | | | Hours. | Number. | | | Summary. |
|---|---|---|---|---|---|---|---|
| Articles. | Articles. | Articles. | | Wet. | Dirty. | Ep. | |
| | | | 8 | | | | No. of patients having fits...... |
| | | | 9 | | | | No. of fits ........ |
| | | | 10 | | | | No. wet ........ |
| | | | 11 | | | | „ dirty ........ |
| | | | 12 | | | | „ raised ........ |
| | | | 1 | | | | |
| | | | 2 | | | | |
| | | | 3 | | | | |
| | | | 4 | | | | |
| | | | 5 | | | | |
| | | | 6 | | | | |
| | | | 7 | | | | |

Attendants late..........................................................

„ absent ..........................................................

Heating of ward and temperature.........................................

Lighting..........................................................

Water ..........................................................

Officers' Visits:

..........................................................

..........................................................

..........................................................

Special Reports:

..........................................................

..........................................................

..........................................................

.......................................... } Night nurses.
..........................................

### To Nurses.

We invite contributions from any of our readers, and shall be glad to pay for "Notes on News from the Nursing World," or for articles describing nursing experiences at home or abroad dealing with any nursing question from an original point of view according to length. The minimum payment is 5s. Contributions on topical subjects are specially welcome. Notices of appointments, letters, entertainments, presentations, and deaths are not paid for, but we are always glad to receive them. All rejected manuscripts are returned in due course, and all payments for manuscripts used are made as early as possible after the beginning of each quarter.

### Everybody's Opinion.

"NATIONAL AND INTERNATIONAL."

MISS L. L. DOCK, Secretary of the International Council of Nurses, writes:—In the interests of journalistic accuracy, which you without doubt desire, I beg to refer to several points in an article of yours of July 16, called "National and International," which, as they now stand, are quite calculated to convey incorrect impressions, and I ask of you the courtesy of inserting the following explanations. In the account of the Berlin meeting, your article conveys the impression that a few isolated individuals were present from England, and it then remarks vaguely, "The United States had a somewhat similar representation." May I supply the information which your article lacks, and say that the representation was precisely similar: the English and Irish delegates represented the Matrons' Council of Great Britain and Ireland and all of the existing self-governing or voluntary associations of English and Irish nurses, and the American delegates represented the American Society of Superintendents of Training Schools for Nurses and the National Associated Alumnæ. In the next paragraph your article says, "It is a misuse of terms to take a small clique of women . . . . and let them arrogate to themselves positions and powers to which they have no right whatever." May I point out that it is only in Russia that individuals are not allowed to associate themselves voluntarily together for common aims and purposes, and could your decree be logically extended it would abolish every voluntary national and international grouping in its early stages? In connection with this idea, but separated by a paragraph, may I refer to the suggestion of your article that the outcome of the Select Committee on Nurse Registration "will probably be the formation of some nursing council, . . . and that council, if formed, should be the real thing," etc.? I will venture to point out that it is quite too late for your article to make suggestions as to the composition of the International Council of Nurses. It is working under a constitution which provides for the affiliation of national bodies of nurses, and the affiliation—not of "some council, if formed," but a council now in process of formation—is promised from England, as well as from Ireland. A considerable error is made in the statement that "this stage army has imposed upon the nurses of the United States," etc. The nurses of the United States, who are informed on international matters and who are supporting affiliation, are not in the least imposed upon. They understand the English situation quite well and are able to discount the opposition to self-government and legal status which comes from would-be managers and financially interested persons, or from caste prejudice. They are prepared to be on friendly terms with all nurses of other countries, as soon as these show their willingness to respond, and any attempt to carry local and temporary animosities across the water will prove perfectly futile. Since the last paragraph of your article is a plain acknowledgment that just a few persons in England are carrying the whole burden of new work, may I remind you of the wise man (I forget his name) who divided people into two classes—those who did the work, and those who grumbled at the way it was done.

[Miss Dock's letter shows that she herself has been deceived as to the facts of the present situation in Great Britain. Otherwise, seeing that the names of all those present at "The International Council of Nurses" in Berlin in June last are well known and available, Miss Dock would not state that the United States representation and the English representation "was precisely similar." The American Society of Superintendents of Training Schools for Nurses and the National Associated Alumnæ include practically the whole of the most knowledgeable and representative superintendents and working nurses of the United States. But when the lists of the Matrons' Council and the other representatives from Great Britain are examined, they are found not to include the most knowledgeable and representative superintendents and working nurses of Great Britain, whose names are conspicuous by their absence. This fact is recognised by and known to the nursing world in this country, and that is why we properly and accurately applied the term "stage army" to the small clique who have marshalled themselves under numerous banners inscribed "Matrons' Council of Great Britain," "The National" or "The International Council of Nurses," and so forth. Miss Dock may be reassured that no one, even in Russia, could object to any small number of ladies so "associating themselves voluntarily together," if it gives them satisfaction or pleasure. Objection is only and properly taken to this "stage army," when it insists upon being regarded as a representative body of nursing opinion and influence. Miss Dock must please understand that THE NURSING COUNCIL, which we had in mind, was not "a stage army" assembled under a banner inscribed the "International Council of Nurses," but one which will in fact represent all nursing interests and include the names of everybody who holds a representative position in the nursing world in Great Britain at the date of its appointment. Miss Dock's letter confirms the view that the "stage army" "has imposed upon the nurses of the United States" including Miss Dock herself. We fancy, too, that Miss Dock must have forgotten the words of the wise man she refers to whose two types of people were workers and obstructives. The "stage army," in so far as it has any influence at all, is obstructive, and for this reason Miss Dock's wise man would no doubt ban it, if his opinion were available.—ED. THE HOSPITAL.]

REGISTRATION PROS AND CONS.

MISS AMY HUGHES writes: May I correct an error in the article "Registration Pros and Cons" which appears in THE HOSPITAL of August 6th? The nurse I mentioned with two years' training in a fever hospital was accepted (not refused) for general work by a private nursing institution, and after a year's service easily obtained another equally responsible post. It is therefore not astonishing she failed to see further nursing education was desirable. The astonishment lies rather in the case with which such imperfect preparation is foisted upon the general public as constituting "a trained nurse." May I add that I do not advocate a "three years" system only, but I ask for sufficient time to be given for a nurse to learn her work thoroughly? All skilled labour requires a term of apprenticeship, and of late three years have been accepted as a time standard for nurses. Registration must be voluntary, as no law can prevent the term "nurse" being applied to one who tends the weak and ailing; but no one should be allowed to pose as a "trained nurse," with all that this implies, who has not satisfied an independent Central Board of the validity of her certificates and the practical knowledge she possesses of her calling.

THE RURAL MIDWIFE AND THE DISTRICT NURSE.

MRS. HEYWOOD JOHNSTONE, Bignor Park, Pulborough, writes: Many inquiries are being made throughout the country as to what steps will be taken through the County Councils to carry out the Midwives Act. It is a very healthy sign that there is a lively anxiety to see that this Act does not fall as a dead letter in the rural districts, but that the people should be habituated to its provisions before the time arrives when it will be penal for any untrained woman to take up the profession of a midwife. The County

Council of Northamptonshire have issued an excellent paper with instructions to their inspectors. They have provided steam disinfectors to insure the proper disinfection of the midwives' clothing after cases of puerperal fever. Indeed all the preparations for the administration of the Act in this area seem from their most interesting report of 1903, most exhaustive. In one County Council area 31s. 6d. is paid for every midwife inspected, and in another 10s. 6d. for every case of puerperal fever reported, and a grant is made for the expenses of clerical work. I have only heard of three instances where direct assistance is given towards the training of midwives out of the funds of the Education Committee, but they are indirectly assisted through the grants to nursing associations who now are adding midwives to their staff. This direct help would, I believe, be a very great assistance, as it is so impossible for some of these women to afford the first expense of training, and where it is done through the charity of the Nursing Associations there is the possibility that these women may be used indiscriminately for general and maternity work. Such a combination is most unadvisable, and from the dangers of which it is the primary object of the Midwives Act to save the lying-in woman. An uneducated "Gamp" may through ignorance bring the infection of dirt to her patient, but the district nurse who goes from cottage to cottage must come in contact with cases that no amount of antiseptic treatment will render harmless to the mother at this time, and therefore the midwife or maternity nurse should never simultaneously be nursing maternity and general cases. What, some will say, are we to do? In our small rural villages we cannot afford two women, and therefore our district nurse must take both. My answer is, enlarge the area of both, and send them further afield to their separate vocations. It could in most cases be easily worked. Do not pauperise the people; let them pay as much as they have paid the untrained women of the past, and you will be surprised to find how far this will go towards covering the expenses, and many too will give a small subscription to help forward this good work, which will save so much suffering and danger to life. Further, I should say, guarantee the doctor a fee if called in, get his assistance and supervision, and you will find the county inspector's work will be light and his reports satisfactory. I wish the Midwives Committee in every area could afford at least one midwife to act under their immediate direction as such, and themselves arrange a moderate payment from the people. It would be a good beginning, and form a standard for others starting on their own account. No doubt the training, if good and thorough, is more expensive than under the old dispensation, and it is necessary the training centres should be in populous places, where there are plenty of births, as otherwise the probationers will not see enough variety of cases. This peremptorily shuts the door on too short a training or too small a lying-in home, as has been the experience of the Rural Midwives Association, 47 Victoria Street, S.W., whose work it is to find and train cottage midwives for those who require them. In connection with what I have already said regarding the profession of the cottage midwife, I will conclude by quoting a letter I have had leave to publish on this point from Dr. Cullingworth, late president London Obstetrical Society and a member of the Central Midwives Board:—"It is most undesirable that nurses whose business it is to go from house to house in district work, attending all sorts of cases and dressing all sorts of wounds and sores, should at the same time be acting as midwives, and coming into close personal contact with lying-in women. The risk in regard to surgical dressings is, in my opinion, greater than in the infectious fevers, and therefore no rule forbidding attendance upon the latter would be a sufficient safeguard. You will easily see, therefore, that, in my view, it should only be under circumstances of the direst necessity that a woman should be permitted to exercise the combined functions of a midwife and an ordinary district nurse. I know the difficulties that arise in the sparsely populated country districts, and the almost impossibility, here and there, of keeping the two sets of duties in entirely different hands. But I am not the less sure that this should always be aimed at, and that any arrangement that does not ensure it should be regarded as a merely temporary measure to be superseded as soon as ever it is practicable."

### Appointments.

Brompton Consumption Hospital, Country Branch.—Miss Spencer has been appointed matron. She was trained at St. Thomas's Hospital, London, and has been night sister, day sister on holiday duty, and sister-housekeeper in the same institution.

Lewisham Union Infirmary.—Miss Henrietta Strick has been appointed matron. She was trained at Leicester Infirmary, where she was afterwards staff nurse. She has since been sister and assistant matron at Lewisham Infirmary.

Portsmouth Parish Infirmary.—Miss Hartley has been appointed night superintendent, and Miss Dickenson and Miss Knight sisters. Miss Hartley was trained at the Bradford Union Infirmary, and has since been sister at the Portsmouth Parish Infirmary. Miss Dickenson and Miss Knight were trained at the Portsmouth Parish Infirmary.

Stroud Hospital.—Miss E. G. Holmes has been appointed matron. She was trained at the Queen's Hospital, Birmingham. She has since been sister at the Birmingham and Midland Ear and Throat Hospital, and ward sister and assistant matron at the Swansea General and Eye Hospital.

The Lady Ampthill Nursing Institute, Madras.—Miss Charlotte Andrews has been appointed nurse. She was trained at the Stapleton Union Hospital, Bristol, where she has since been charge nurse. She holds the London Obstetrical Society's certificate.

Victoria Memorial Jewish Hospital, Cheetham, Manchester.—Miss Hotson has been appointed matron. She was trained at the Sussex County Hospital, Brighton, and has since been charge nurse at Sussex County Hospital and at Rawson Convalescent Home, Harrogate; matron of the Miners' Hospital, Skelton-in-Cleveland; charge nurse at the Mariners' Homes, Egremont, Cheshire; and temporary charge nurse at the Princess Mary Homes, Addlestone. She has also done private-nursing for several years.

West End Hospital for Diseases of the Nervous System, London.—Miss Rosa I. Honeyman Brown, the assistant matron, has been appointed non-resident head masseuse in place of assistant matron.

Wolverhampton and Staffordshire General Hospital.—Miss A. Stuart Cameron and Miss Eleanor E. Meadows have been appointed sisters. Miss Cameron was trained at the Royal Infirmary, Dumfries, and has since been sister at Wrexham Infirmary, and night sister at the Royal Infirmary, Preston. Miss Meadows was trained at Wolverhampton General Hospital, and has since been receiving-room nurse in the same institution.

### Death in Our Ranks.

We regret to hear of the death, from typhoid fever, of Miss Kattie Fleming, a probationer at the Royal Devon and Exeter Hospital. Nurse Fleming, who passed away on Thursday last week, and was buried on Saturday in Exeter Higher Cemetery, was a native of Waterford, and her father, brother, and two sisters were present at the funeral, as well as the matron and twenty-six members of the hospital nursing staff.

### Wants and Workers.

The Editor of The Hospital will be glad if any readers who have spare copies of the journal for January 20th, February 10th, March 10th, June 30th, and August 25th, 1900, will communicate with the Manager, Scientific Press, 28 Southampton Street, Strand, W.C.

### A Book and its Story.

MR. BENSON'S NEW NOVEL.*

Mr. Benson's new book, "The Challoners," is very interesting, and at the same time very disappointing. He is seen at his best, as usual, in vivid scenes in which the people and the dialogue are perfectly natural. But his weakness is shown in the failure to face a psychological problem raised by the strife of principles, versus temperament, in the characters of Mr. Challoner, the rigid, conscientious clergyman, and his twin children Helen and Martin. The final chapters suggest that, having worked up the subject to a given point, the author grew weary of it, and hurried it to an abrupt conclusion, but in spite of these defects the book is very readable. The Honourable and Reverend Sidney Challoner held a country living. He had married as a young man a pretty girl who was half Italian by birth, and she died when the twins were born. They were now grown up, and Martin was at Cambridge. Between the children and their father lay a wall of reserve which caused the secret tragedy of his life. We read: "He longed for the ordinary human affections and relationships with the same intensity with which he served God, but through the armour crust of his nature, an armour be it noted, of welded and hammered work and duty, his human hand could not break its way to clasp the hand of others." And so this father, who loves his motherless children deeply, fails through sympathy with their alien temperaments to make allowance for things which, wholly absent from his own, could be traced to the maternal influence only. For this he made no more allowance than he had done for his girl wife, whose motor power in life had been joy. "From the first the marriage had been strangely ill-assorted; it may have been made in heaven, but in that case it would probably have been far better if it had not come down to earth." A year after their marriage the twins had been born, and from that time the girl-mother had drooped and dwindled. The fogs of this northern climate, fogs, too, more intimate and distressing of mind and spirit, the absence of mirth and laughter, chilled her to the bone, and a year afterwards she was dead." Of her husband's character the following extract will serve as an outline to suggest the causes which led to the stern exterior. "His study where he worked was singularly like himself, and seemed as integral a part of him as the snail-shell is of the snail. . . . a scrupulously orderly and energetic severity, in fact, was the key-note of the room. . . . He was master of a style of English in itself neat, correct, and lucid, which served him, not as in the sermons of so many preachers, to clothe and cover his lack of ideas, but to reveal the abundance of them. There survived in him, indeed, a full, if not double, portion of the Puritan spirit on religious matters; and though his mind, his soul, his actions, were all dictated and impelled by a fervent and whole-hearted Christianity, yet his eloquence was wont to dwell, and did so here (he was writing a sermon on the less encouraging and comfortable verses in the Athanasian Creed) on the doctrine of eternal damnation with a very curious gusto. The Puritan, too, survived in a certain mistrust he had of mirth and gaiety; without being in the least sour, he was so intensely serious that at any given moment it appeared to him that there was probably something better to do than to laugh, and a moment's thought easily discovered what it was. Of work he was insatiable; if he was unsparing to others, at any rate he never spared himself, and the day of rest was to all in his house the most iron day of all." His daughter Helen was chaperoned by an unmarried sister of his own, who was "one of those not uncommon English spinsters who have a perfect passion for doing the things she ought to do and for leaving undone the things she ought not. As the feminine element in the house of a pariah priest it was her clear mission to be aunt, if not mother, of the flock." The classes and instructions incident to her position were fortunately undertaken willingly and thrived successfully, for her niece, Helen, performed the lesser part of the parochial duties allotted to her without active interest or pleasure in them. Helen is described on the opening page: "She wore no hat, and the dusky smouldering gold of her hair lay low over her forehead. Her eyelids, smooth with the unwrinkled firmness of flesh of twenty-two years, drooped low over her book, but between the lids there showed a thin line of matchless violet." She was reading, and the book was "The Mill on the Floss," "a book by an author who, however celebrated, yet remained in the opinion both of Helen's father and aunt a person of unchristian belief and heathenish conduct. As she read on, "her half-smoked cigarette dropped from between the fingers of the left hand, and sent little whorls of blue smoke as it lay unheeded on the grass, and her eyes grew suddenly dim. Then the last page was turned, and with a sudden sobbing intake of breath she closed the book." This morning her father had had a more than usually unpleasant talk with her brother Martin on the subject of his late failure at Cambridge, proceeding not from lack of ability, but from a general slackness which was found in everything, to which his mind should have been given, save one—music. Here was concentrated all his mental energy and to this pursuit, upon the persuasion of many influential friends, his father finally allowed him to devote himself. Then Helen becomes engaged to Lord Yorkshire, a neighbouring landowner, who after being accepted acknowledges himself an atheist. He is spoken of as "a pleasant-faced young man, who was rather stout, with smooth flaxen hair and rather prominent blue eyes."

The humour of the book is supplied by the dialogues of Lady Sunningdale, who suggests "Dodo" in maturity. "Years were the only thing in which Lady Sunningdale was no longer young; but the youthfulness of her mind, tastes, character, was spontaneous and natural, and she still retained to the full all the eager curiosity of youth, all youth's insatiable capacity for pleasure. In person she was very tall and largely made, but she moved with an exquisite briskness and vigour, and though stout still clung to her waist. She had few habits, and was thus very adaptable, but one was to make a punctual first appearance before luncheon. . . . She seldom walked except at this stated time, but she made up for her lack of walking by talking; this she did on all occasions to as many persons as possible, and was extremely entertaining." Her conversation flows on with a delightful and inconsistent discursiveness, undeterred by reflections as to its effect on the listener. She was staying "for rather an extended week-end" with Lord Flintshire, the rector's brother. Turning to him she exclaims, "Yes! isn't it a divine morning? Why is one such a fool as ever to leave the country and go to London? If one had a single spark of originality one would never go near it. Yes, please put up my sunshade frame. I know I look hideous this morning; but it does not matter how one looks in the country, which is another of its charms. But I didn't sleep a wink; I never close my eyes in the country; really, London is the only place to live in. I have contradicted myself, have I not? Who cares? I'm sure I don't. . . . It shows, in fact, that one's powers of sympathy and of seeing other points of view are defective unless one sees both sides of every question and upholds both vehemently." She quotes, *à propos* of someone she has in mind, "Beauty is probably evil in its origin, which accounts for the extreme plainness of good people."

"The Challoners" ends when it was becoming really arresting and without the solution of more than one point of interest. Perhaps Mr. Benson intends to leave this for another book.

* "The Challoners." By E. F. Benson. Heinemann. 6s.

### Echoes from the Outside World.

### Movements of Royalty.

An incident of the Cowes week was that on Thursday the Kaiser's yacht, the *Meteor*, competed in a race. Just after she had taken in her jib foresail an extra strong capful of wind caught the yacht. The strain proved too much for the mast-head strop to which the main halyard block is attached, and the huge mainsail collapsed quickly to the deck, almost at the feet of the King, who was standing aft on the weather quarter. His Majesty saw what was coming, and moved back a little. The accident, which happily had no ill results, is attributed to one of the American inventions with which the *Meteor* is furnished. The Royal party afterwards remained on deck during the progress of a thunderstorm of considerable violence. On Saturday the King and Queen were present at a military torch-light tattoo, given at Carisbrooke Castle by the 2nd Sherwood Foresters. The tattoo was carried through with complete success on the bowling-green, and their Majesties congratulated the commanding officer on the success and smartness of his men's performance. The scene of the tatoo, with the walls of Carisbrooke Castle and Keep ablaze with torches, and the bright scarlet uniforms of the military, was brilliant. The soldiers with their torches finally formed on the bowling-green to represent the Royal initials, E.R., and in this formation they sang the first and last verses of the hymn, "Abide with me," accompanied by the band. On Monday their Majesties returned to London. A large crowd assembled at Victoria Station to witness the arrival of the Royal party. The King and the Queen, with Princess Victoria, drove at once without escort to Buckingham Palace in an open landau, and the Prince of Wales, in a closed carriage, to Marlborough House. On Tuesday the King and Queen were the recipients of many congratulations on the occasion of the second anniversary of their Coronation. They spent the day quietly at Buckingham Palace, where they were visited by the Prince of Wales and other members of the Royal Family. Loyal demonstrations took place in India, Windsor, Portsmouth, and elsewhere. On Wednesday the King left town for Marienbad, and the Queen for Sandringham.

### British Force at Lhasa.

It is officially announced that the British force under Brigadier-General Macdonald arrived at Lhasa on Wednesday, August 3rd, and is camped about a mile from the Grand Lama's palace, the Potala, and in the immediate vicinity of the Dalai Lama's private garden. The force was met at some distance from the city by the representative of the Nepaulese Resident, who said that a faction in Lhasa had sworn rather to die than to let the British troops enter the place, but that the delegates had intimated, by beat of drum, that there must be no fighting, as the Tibetans there would be as dust beneath the British feet. The faction then dispersed. When the camp had been pitched the Amban paid Colonel Younghusband a ceremonial visit and expressed himself willing to assist in arriving at a settlement.

### The Viceroy of India.

The King, it is announced, has approved the reappointment of Lord Curzon of Kedleston to be Viceroy and Governor-General of India. It is not every one who is aware that as soon as Lord Curzon left Indian soil he ceased, by law, to be the Viceroy. The state of his health rendered it essential that he should enjoy a holiday before he entered upon a second term of office, and Lord Ampthill was therefore appointed Viceroy for the time being. Lord Curzon has now perfectly recovered, and will return to India on September 30th in order to resume the exalted position he abandoned in the spring.

### The War in the Far East.

According to an official telegram from Tokio, dated Friday last, the Japanese casualties in the attack on To-neuch-cheng amounted to 194 killed and 666 wounded. The Russian losses are estimated at 2,000, and the Japanese captured six field-guns, large quantities or grain and flour, and 33 prisoners. In the engagement at Yushuling-tsu and Yangtsuling the Japanese casualties amounted to 946, including 40 officers, and the Russian losses on this occasion also were estimated at 2,000. About 700 of the latter were left dead on the field. General Stössel has reported from Port Arthur that the garrison repulsed all the Japanese attacks on July 26, 27, and 28, with immense losses. The fleet, he states, assisted in the defence by bombarding the Japanese flank. The Russian losses during the three days were 1,500 men and 40 officers killed and wounded. At Tokio it is denied that the Japanese losses were heavy.

### Death of Lady Tweedmouth.

The short illness of Lady Tweedmouth terminated fatally on Friday night at her Highland home in Inverness-shire. She was only 51 years of age. Before her marriage in 1873 she was Lady Fanny Churchill, her five sisters being Lady Wimborne, the Dowager-Duchess of Roxburghe, Lady de Ramsey, Lady Howe, and Lady Sarah Wilson. The late Lord Randolph Churchill was, of course, her brother, and Mr. Winston Churchill is a nephew. Unlike most of her sisters her political sympathies were with the Liberals, and when she married Mr. Edward Marjoribanks, subsequently Lord Tweedmouth, she became well known in the social world of Liberal politics, using her utmost exertions to render the Liberal Social Council an organisation of influence. During the Boer war she assisted in the work of the Victoria League, and her domestic interests were numerous and varied.

### Train Disaster Caused by a Cloud-burst.

An extraordinary railway accident is reported from America, similar to the Tay Bridge disaster in December 1879. While the Missouri Pacific express from Denver was running over the Denver Rio Grande line on Sunday evening, the locomotive and three cars fell through a bridge near Eden, eight miles from Pueblo, Colorado. The engine was almost over the bridge when it collapsed. Two sleeping-cars remained on the rails, but the smoking and baggage-cars and the chair-car were carried miles down the creek by the rushing water. The express-car has been found near the scene of the wreck with the safe open and its contents gone. A great number of volunteers are searching for the bodies of the killed, but it is probable that a complete list will never be published, as both the conductor and the engine-driver are among the victims. Throughout Southern Colorado there has been one of the heaviest rain and windstorms known for years, and two rock-slides are reported at other points on the line. It was this cloud-burst that converted an ordinarily dry creek into a raging torrent, which weakened the foundations of the bridge.

### "Earl's Court" of 1905.

It is officially denied that this is the last year during which an Exhibition will be held at Earl's Court. The show next year will be called the Naval, Shipping, and Fisheries Exhibition, and is intended to represent the naval and mercantile marine, sea and river fishing, and yachting. The Austrian Exhibition has been postponed until 1906 at the request of the Austrian Government.

### Notes and Queries.

**FOR REGULATIONS SEE PAGE 143.**

*Guild of St. Barnabas.*

(142) Kindly give me address where to apply for rules of Guild of St. Barnabas for nurses.—*C. P.*

Write to the general secretary, Miss C. J. Wood, The Nurses' Hostel, Francis Street, London, W.C.

*Stuffing.*

(143) Can you tell me what is the best stuffing for a pillow for babies that perspire greatly? Is chaff?—*A. R.*

Excess of perspiration in children is usually a sign of rickets. A medical man should be consulted.

*Work.*

(144) Can you tell me of any institution which would find work for auxiliary nurses? I have had much difficulty in finding employment, as I left London 18 years ago in order to nurse my own people.—*Nurse H.*

Write to the Auxiliary Nurses' Society, 10 Orchard Street, W.

*Private Nursing.*

(145) (1) Can you give me the name of a book on private nursing? (2) When a nurse begins working on her own account is it better to draw up a list of rules as to hours of duty, etc., and give it to her patients?—*Nurse H.*

(1) "Hospital and Private Nursing." By Miss S. E. Orme, published by the Scientific Press, 1s. (2) A private nurse cannot make rules for her patients.

*Riviera.*

(146) Can you possibly supply me with the addresses of nursing institutions on the Riviera? I enclose a stamped envelope, etc.—*S. H.*

A fee of 2s. 6d. should accompany any query requiring a private reply. The English Nursing Institute, Avenue de la Gare, Mentone; the Nice Nursing Institute, Avenue Désambrois, Nice; the English Nurses' Institute and Medical Home, Sunnybank, Via Borgo Pescio, San Remo, are the chief.

I am wishing to go to Switzerland, Italy, or the South of France next winter. Will you kindly give me the names of any nursing institutions in those places?—*M. E.*

See reply to *S. H.* Also there are the English Nurses' Home, Villa Albany, Biarritz; the Davos Invalid Home, Davos Dorf, Switzerland; the Association of Trained Nurses and Masseuses, 7 Via Rondinelli, Florence; the Anglo-American Nursing Home, 256 Via Nomentana, Rome; and the St. Paul's Home for Nurses, 25 Via Vicenza, Rome.

*Massage.*

(147) Will you kindly tell me where it would be best for me to learn massage? I have been a trained nurse now for three years.—*H. M. P.*

Write fully to the Secretary, the Incorporated Society of Trained Masseuses, 12 Buckingham Street, Strand, W.C.

Please inform me of the best possible way to obtain instruction in massage. Can I obtain such by becoming a male probationer in the National Hospital for the Paralysed and Epileptic?—*J. C.*

Certainly.

*Metropolitan Hospital Sunday Fund.*

(148) Will you kindly give me the address and tell me to whom I should apply for a donation from the Metropolitan Hospital Sunday Fund?—*E. T., Matron.*

Address the Secretary, Metropolitan Hospital Sunday Fund, 18 Queen Victoria Street, London, E.C.

*Bunion.*

(149) I should be glad to know of a cure for a bunion; it is so very painful in spite of the fact that I have had my shoes made large in order to avoid all pressure on the joint.—*Sister G.*

Yours is a case for medical advice.

**Important Nursing Text-books.**

"The Nursing Profession: How and Where to Train." 2s. net; 2s. 4d. post free.

"The Nurses' Dictionary." By Honnor Morten. 2s. post free.

"Nursing: a Complete Text-book of Medical, Surgical, and Monthly Nursing." By Percy Lewis, M.D. 3s. 6d., post free.

"Elementary Physiology for Nurses." By C. F. Marshall, M.D. 2s. post free.

"A Complete Handbook of Midwifery for Nurses." By J. K. Watson, M.D. 6s. net; 6s. 4d. post free.

The above works are obtainable of all booksellers, or direct from The Scientific Press, Limited, 28 & 29 Southampton Street, Strand, London, W.C.

### For Reading to the Sick.

SOLACE IN SOLITUDE.

O ye in solitude who weep,
Whose souls a sorrowing vigil keep;
Whose hearts are breaking with the strain—
Striving true gladness to attain;
Know that by night as well as day
God wipeth every tear away.

By painful process and by slow
The rootlets of our spirits grow;
Till strengthened by the toil and strife
They burst in bloom of truest life:
Love twines her tendrils round our grief,
And brings the stricken heart relief.

*Wm. G. Kingsland.*

A time of sickness is a grace that goes to make us kind and merciful. Others are kind to us then, and we must repay in the same golden coin. My God, help me to learn this lesson to the fullest extent, that, while I know how much I need Thy mercy, I may be merciful and gentle to all others in thought, word, and deed. If we wish for the mercy of God we must show mercy to others.

Day by day goes by, and others must wait on me when I would gladly wait on them.

But stay awhile: is this really true? When our Lord passes by your home night after night, begging an alms and seeking fruit, do you answer from within: "Pass on, for I have nothing to offer: I have been in bed all day and have done nothing for you, My Lord?" No; you cannot say this. The poor Man at the door is crowned with thorns, laden with a cross, weary and thirsty, and when to Him you hold out the twelve hours and more of pain which you have borne that day for Him and for His interests, He receives it all with joy. He gives you His blessing and says only two words: "Well done." And Jesus of Nazareth passes by, leaving you at peace, saying He will call next day again and meanwhile will not forget you, and that one day He will call to take you with Him to one of the mansions prepared for you, and which you have adorned by many days of patient suffering.

Pain shall teach you your dependence on others, on our Lord first, and then on your friends. It will show you the beauty of charity, the value of sympathy, the power of kindness, the need and relief of one or more trusted friends. This lesson will help to make you gentle to others, free from selfishness, always kind. It will help you to understand the love of the sacred heart for you, to gauge the beauty of its "new commandment," "to love one another" and "to bear one another's burdens." Leave the school of pain, if it be God's will, but never forget what you have learnt there.—*Anon.*

Leaving the final issue in His hands
Whose goodness knows no change, whose love is sure,
Who sees, foresees, who cannot judge amiss.

